# Simulators of Squamous Cell Carcinoma of the Skin: Diagnostic Challenges on Small Biopsies and Clinicopathological Correlation

**DOI:** 10.1155/2013/752864

**Published:** 2013-06-25

**Authors:** Kong-Bing Tan, Sze-Hwa Tan, Derrick Chen-Wee Aw, Huma Jaffar, Thiam-Chye Lim, Shu-Jin Lee, Yoke-Sun Lee

**Affiliations:** ^1^Department of Pathology, Yong Loo Lin School of Medicine, National University Health System, National University of Singapore, Lower Kent Ridge Road, Singapore 119074; ^2^University Medicine Cluster, National University Health System, Singapore 119074; ^3^University Surgical Cluster, National University Health System, Singapore 119074; ^4^Departments of Otorhinolaryngology and Hand and Reconstructive Microsurgery, National University Health System, Singapore 119074

## Abstract

Squamous cell carcinoma (SCC) is a common and important primary cutaneous malignancy. On skin biopsies, SCC is characterized by significant squamous cell atypia, abnormal keratinization, and invasive features. Diagnostic challenges may occasionally arise, especially in the setting of small punch biopsies or superficial shave biopsies, where only part of the lesion may be assessable by the pathologist. Benign mimics of SCC include pseudoepitheliomatous hyperplasia, eccrine squamous syringometaplasia, inverted follicular keratosis, and keratoacanthoma, while malignant mimics of SCC include basal cell carcinoma, melanoma, and metastatic carcinoma. The careful application of time-honored diagnostic criteria, close clinicopathological correlation and a selective request for a further, deeper, or wider biopsy remain the most useful strategies to clinch the correct diagnosis. This review aims to present the key differential diagnoses of SCC, to discuss common diagnostic pitfalls, and to recommend ways to deal with diagnostically challenging cases.

## 1. Introduction

Squamous cell carcinoma (SCC) is amongst the top 3 common skin cancers, ranking behind basal cell carcinoma (BCC) and ahead of melanoma [1]. It is a tumor that is locally invasive and which has the capacity to metastasize. Prognostically, it also occupies an intermediate position between BCC and melanoma, with BCC being locally invasive but typically nonmetastasizing, while melanoma having the well-known capability to metastasize. Histopathologically, most cases of SCC are readily diagnosable. However, diagnostic challenges are occasionally encountered and contributed mainly by the myriad of histopathologic mimics of SCC and small biopsies that sample only part of the lesion [2]. 

For the simulators of SCC, on the one hand, there are benign squamous lesions that appear to be infiltrative histopathologically. On the other hand, there are other malignant skin tumors that may display squamous differentiation or which elicit squamous proliferation that mimic SCC. Misdiagnosis of benign lesions as SCC would result in unnecessarily extensive surgery, while delayed diagnosis of SCC could lead to local tissue destruction by tumor, sometimes metastatic disease, and even death. Some malignant differential diagnostic entities, for example, melanoma and Merkel cell carcinoma (MCC), have worse prognosis or require different surgical strategies and differ in the need for adjuvant treatment [3, 4]. This review will present all the salient benign and malignant differential diagnoses of SCC, highlight diagnostic pitfalls, and suggest strategies for clinching the appropriate diagnosis.

## 2. Benign Squamoproliferative Lesions That Mimic SCC

### 2.1. Inverted Follicular Keratosis

**Figure 1:**
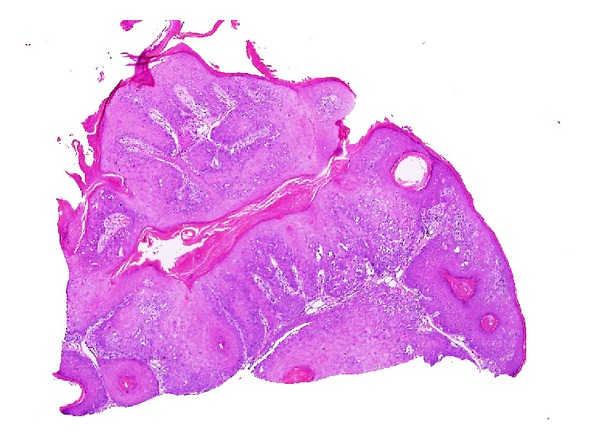
Inverted follicular keratosis: lesion shows proliferative downgrowths of mature squamous epithelium with infundibular keratinization (H&E ×40).

**Figure 2:**
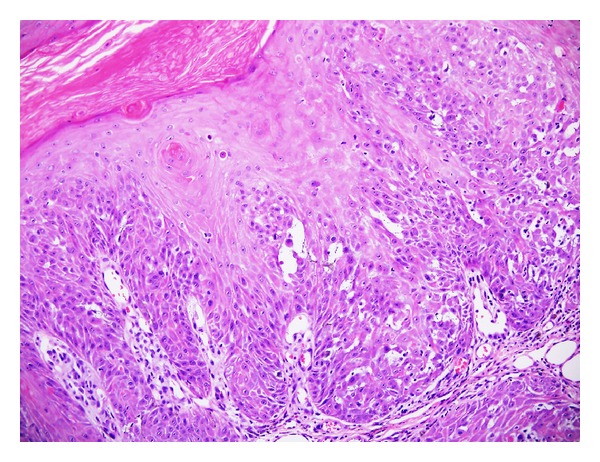
Inverted follicular keratosis: higher magnification showing squamous eddies (H&E ×200).

Inverted follicular keratosis is a lesion that histopathologically shows downgrowths of follicular squamous epithelium and the adjacent epidermis [5]. It commonly occurs in middle-aged and elderly patients, presenting as a scaly papule, nodule, or plaque. It morphologically and conceptually overlaps with irritated seborrhoeic keratosis. In contrast to SCC, it has circumscribed borders, and significant cellular atypia is not seen (Figures 1 and 2).

**Figure 3:**
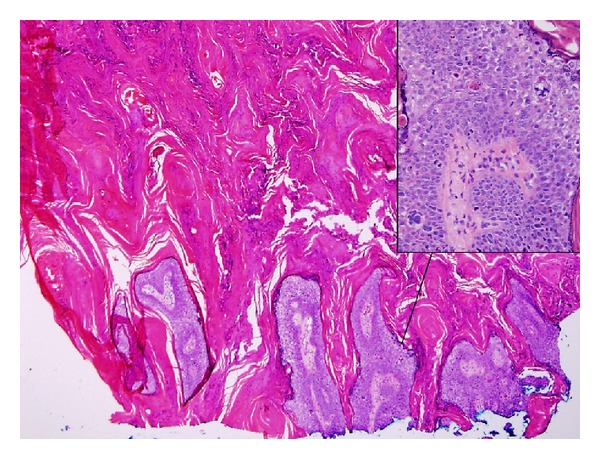
Bowen disease: Shave biopsy specimen showing mainly papillomatous epidermal lesion with hyperkeratotic horn. Inset: closer view of the underlying lesional epidermis shows cells with nuclear pleomorphism, prominent nucleoli, and frequent and abnormal mitotic figures. (H&E, ×20; Inset: ×400).

A pitfall with regard to differential diagnosis of a supposedly benign squamous keratosis is when encountering a specimen of a superficial shave. Histologically, attention should be turned away from the hyperkeratotic superficial horn to the underlying lesional epithelium, which may be limited in amount in the specimen (Figure 3). The presence of significant atypia or abnormal maturation would raise the possibility of actinic keratosis or Bowen disease or even the superficial aspect of an invasive SCC.

### 2.2. Pseudoepitheliomatous Hyperplasia

**Figure 4:**
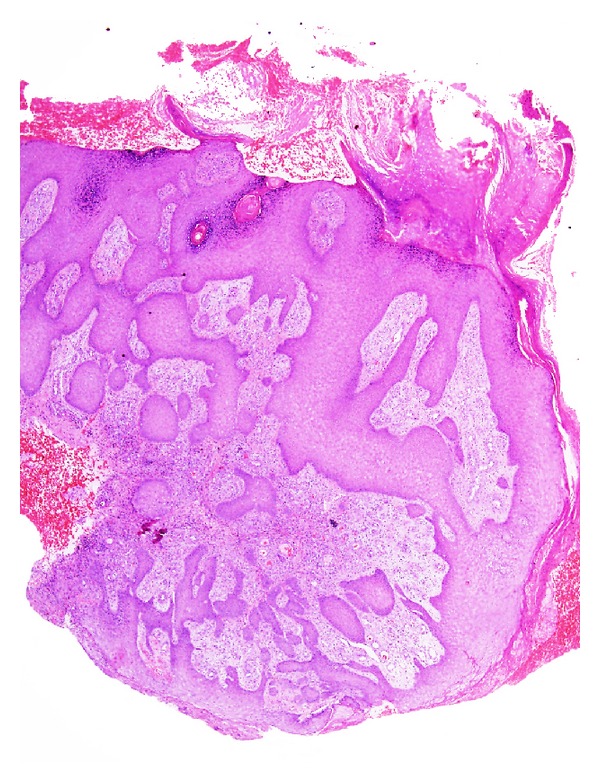
Pseudoepitheliomatous hyperplasia featuring acanthotic squamous epithelium showing irregular thick finger-like downgrowths into the underlying dermis. (H&E, ×20).

**Figure 5:**
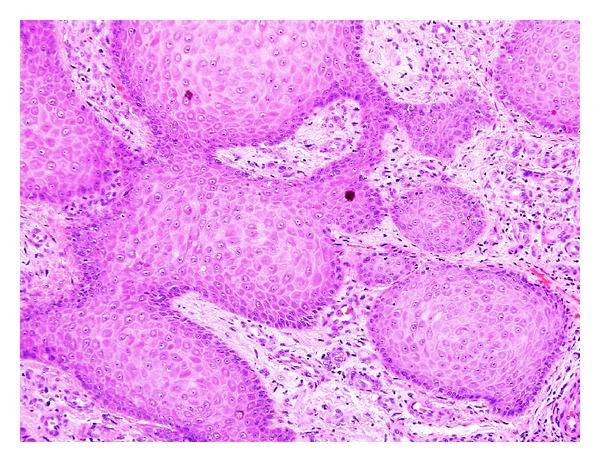
Pseudoepitheliomatous hyperplasia: higher magnification view showing reactive-appearing squamous downgrowths with no significant cytologic atypia. The dermis shows mild chronic inflammation and granulation tissue formation (H&E, ×200).

Pseudoepitheliomatous hyperplasia (PEH) is a florid proliferation of the epidermis that may occur in the setting of chronic skin ulcers, abscesses, burns, and infections [6]. PEH with neutrophilic microabscesses is typically seen in halogenodermas, infections like chromomycosis and blastomycosis, granuloma inguinale, leishmaniasis, and pemphigus vegetans [7]. While the squamous epithelium of PEH can appear infiltrative, resembling well-differentiated SCC, careful attention should show that marked cellular atypia and abnormal mitotic activity are absent (Figures 4 and 5). A careful search for an inciting infective process should be carried out with examination of deeper levels and use of microbiological special stains. In challenging cases, close clinical correlation and followup are required; rare cases require repeat biopsy for evaluation.

### 2.3. Infundibulocystic Hyperplasia

**Figure 6:**
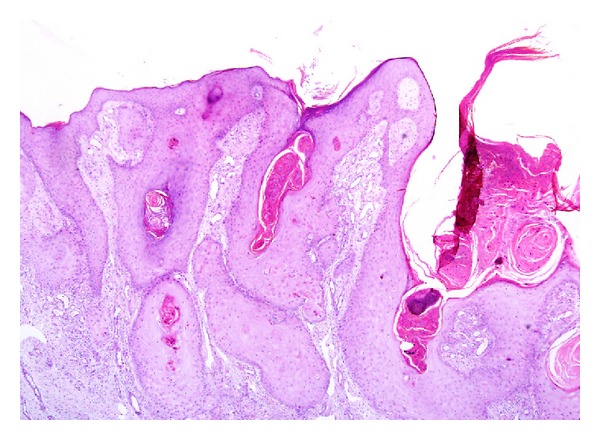
Infundibulocystic hyperplasia: skin lesion showing follicular proliferative process with bland squamous cells and formation of dilated canals containing keratotic material. (H&E, ×40).

**Figure 7:**
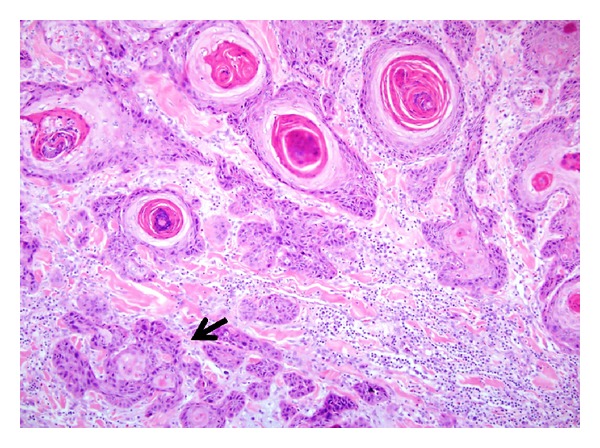
Infundibulocystic SCC: superficial areas showing infundibulocystic canals with deeper infiltrative squamous cell clusters featuring cellular atypia and mitoses (arrow). (H&E, ×100).

Infundibulocystic hyperplasia is a distinctive follicular epithelial proliferative process with formation of dilated canals containing keratotic material [8]. Presenting clinically as a nodule or plaque, it features histologically bland lesional squamous cells and a relatively superficial noninfiltrative deep edge (Figure 6). When a short clinical evolution is noted and the lesion has a crateriform architecture, the features relate more to keratoacanthoma (*vide infra*). When marked cellular atypia or abnormal mitosis is noted, or if deeply infiltrative tongues are present, the lesion should be regarded as malignant, and an appropriate designation is infundibulocystic SCC (Figure 7) [8].

### 2.4. Verrucous Hyperplasia

**Figure 8:**
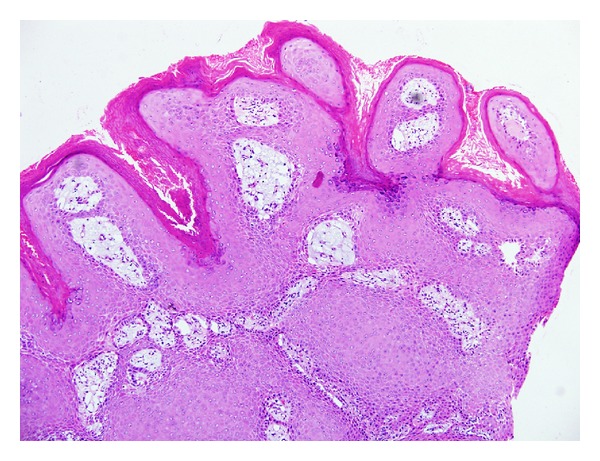
Verrucous hyperplasia: low power magnification view showing epidermal squamous proliferation with broad and superficial downgrowths of the epidermis. Overlying hyper-and parakeratosis is present. There is no atypia or koilocytes. (H&E, ×100).

Verrucous hyperplasia is another type of epidermal squamous proliferation typified by broad and superficial downgrowths of the epidermis (Figure 8). Overlying hyper- and parakeratosis are present. Atypia and koilocytes are absent. This condition usually occurs in the skin within and around the external genitalia and the oral cavity, in reaction to a variety of chronic irritative etiologies. It is distinguished from verrucous carcinoma, which has deeply pushing downgrowths. This feature can be difficult to assess in superficial or poorly oriented biopsies. Nevertheless, verrucous hyperplasia is well known to occur in close proximity, or to precede verrucous carcinoma [9]. Its presence in the context of a clinically worrying lesion should prompt close followup or a deeper and wider biopsy.

### 2.5. Eccrine Squamous Syringometaplasia

**Figure 9:**
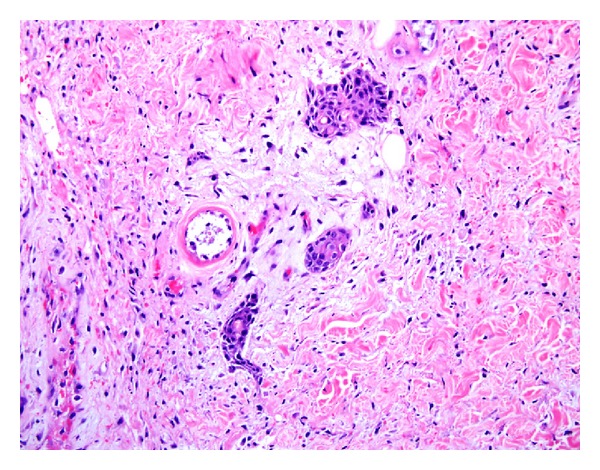
Eccrine squamous syringometaplasia: bland appearing squamous islands in the dermis centered around eccrine lumina. Scattered lymphocytes are present in the surrounding dermis. (H&E, ×200).

Eccrine squamous syringometaplasia (ESS) is a reactive process characterized by squamous metaplasia of eccrine ducts and glands [10, 11]. It commonly occurs in the axillary and groin regions and presents as nodules and plaques. EES is most well described as an adverse drug reaction, mostly to chemotherapeutic drugs [11]. It also occurs in the vicinity of burns, ulcers, and scars [10]. While it should not be confused with SCC clinically, it could cause some confusion histologically. The lesion features cytologically bland squamous islands in the dermis that are closely associated with eccrine lumina and conforming to a lobular configuration (Figure 9). An associated chronic inflammatory infiltrate is usually present.

### 2.6. Desmoplastic Adnexal Tumors

**Figure 10:**
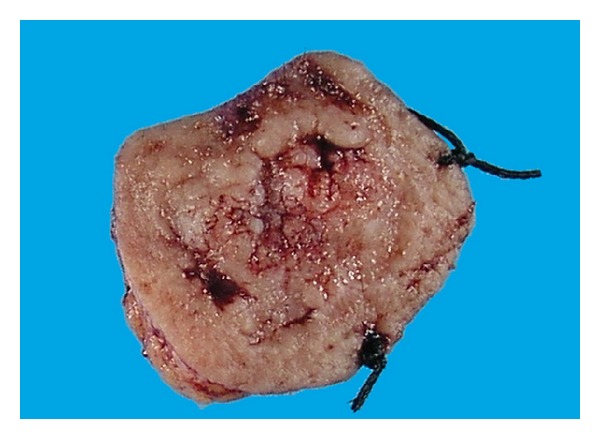
Desmoplastic trichoepithelioma: macroscopic picture showing an ulcerative nodular lesion with raised rolled edges features which mimic SCC or BCC.

**Figure 11:**
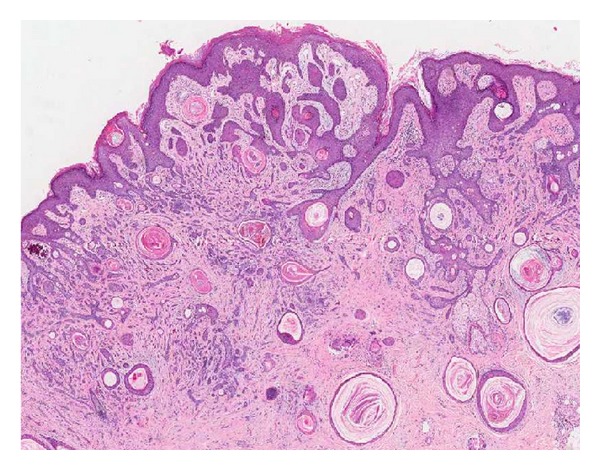
Desmoplastic trichoepithelioma: low power magnification showing hyperplastic downgrowths of epidermis with cords of basaloid tumor cells arranged in a pseudoinfiltrative pattern. (H&E, ×40).

Some benign adnexal tumors may superficially resemble SCC. Desmoplastic trichoepithelioma presents clinically as an enlarging nodule and shows cords and nests of basaloid cells with horn cyst formation, embedded within a sclerotic dermal stroma (Figures 10 and 11) [12]. The overlying epidermis is often hyperplastic. On a superficial biopsy, the hyperplastic downgrowth of the epidermis and the cords of basaloid tumor cells give a pseudoinfiltrative appearance mimicking the features of SCC. The reassuring absence of significant atypia and the paucity of mitotic activity should be sought for. 

Desmoplastic tricholemmoma is another adnexal tumor that can mimic SCC. The cohesive tumor cells growing as cellular sheets or cord-like manner streaming within a sclerotic dermis can appear concerning [13]. Squamous differentiation may also be present. Once again, the lesion should show cytological blandness. Areas of regular tricholemmoma disclosing epithelial cells with clear cytoplasm can usually be found. 

## 3. Benign Nonsquamous Lesions That Mimic SCC

In unusual clinical contexts, benign lesions of varied nature may mimic SCC both clinically and histopathologically.

### 3.1. Extracranial Meningioma

**Figure 12:**
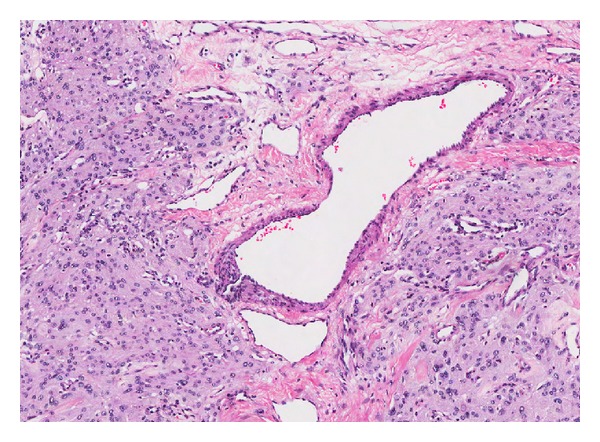
Meningioma: medium power magnification showing syncytial sheets and whorls of tumor cells with bland nuclear features. The presence of nuclear pseudoinclusions, psammoma bodies and the absence of atypia allow the distinction from SCC. (H&E, ×200).

Meningioma has been known to occasionally be located outside the intracranial location to involve the scalp [14]. Histopathologically, the syncytial sheets or whorled arrangement of tumor cells may impart a squamoid appearance (Figure 12). The presence of nuclear pseudoinclusions, psammoma bodies and the absence of significant atypia, in most cases, should allow distinction from SCC. Immunohistochemistry is of limited value in distinguishing the entities as both meningioma and SCC share expression of EMA, and the former is sometimes reactive for cytokeratins as well. 

### 3.2. Decidualized Endometriosis

**Figure 13:**
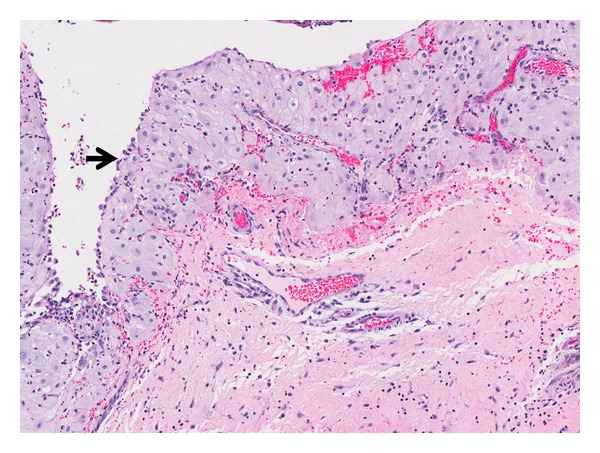
Endometriosis with decidualization: medium power magnification showing cystic space surrounded by large polygonal cells with basophilic cytoplasm and bland nuclei. The cyst wall is rimmed by a layer of endometrial cells (arrow). (H&E, ×200).

Another form of lesional tissue that may sometimes cause diagnostic confusion is decidualized endometrial tissue occurring outside the confines of the female genital tract [15]. The most common context of this is endometriotic deposits in the skin and soft tissue of the abdominal wall or perineum, where background therapy with progestational agents causes decidualization of the endometrial stromal component (Figure 13). Such decidualized cells are plump and form sheets, complete with well-defined intercellular borders, simulating SCC, if not for the notable absence of nuclear atypia. 

## 4. Others Squamoproliferative Tumors That Mimic SCC

### 4.1. Keratoacanthoma

**Figure 14:**
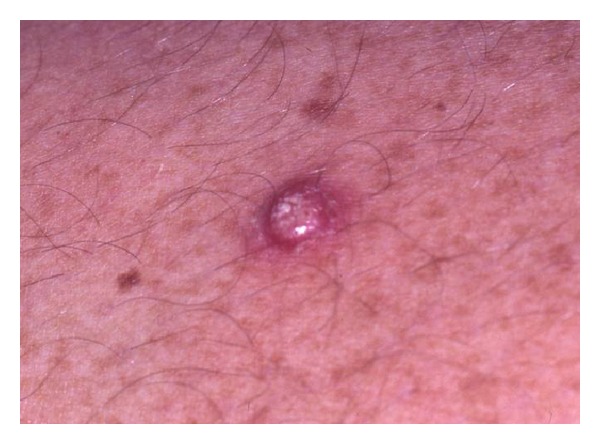
Keratoacanthoma: clinical photograph showing a well-circumscribed reddish nodule with erosion on the surface.

**Figure 15:**
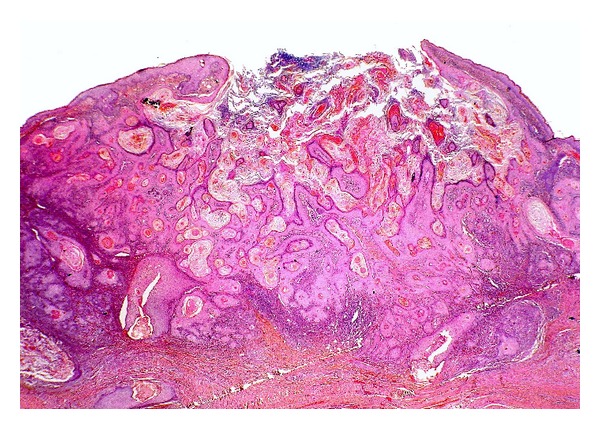
Keratoacanthoma: low power magnification showing a crateriform lesion which is filled with central keratinous plug and bounded by proliferative lesional squamous epithelium. (H&E, ×20).

KA is a well-differentiated squamoproliferative tumor, with a fairly rapidly growing clinical course (Figure 14) [16]. Some authorities consider it a variant of SCC, while others regard it as a unique entity in view of its typically self-involuting course. Histopathologically, it features a cup-shaped or crateriform architecture, containing a central keratinous plug, bounded by lobules of lesional squamous epithelial cells (Figure 15). Features that favor a lesion as a KA rather than being an SCC include a limited pushing deep front, distinct tumour-stromal interface, and lesional squamous cells with abundant cytoplasm centrally. There is usually absence of stromal desmoplasia and of significant cellular atypia [17]. While there have been numerous studies investigating the differences between KA and SCC of the molecules involved in the regulation of cellular proliferation, apoptosis and the cell cycle, the histomorphology, as well as consideration of the clinical tempo of the lesion are still the most practical aspects in routine diagnosis.

**Figure 16:**
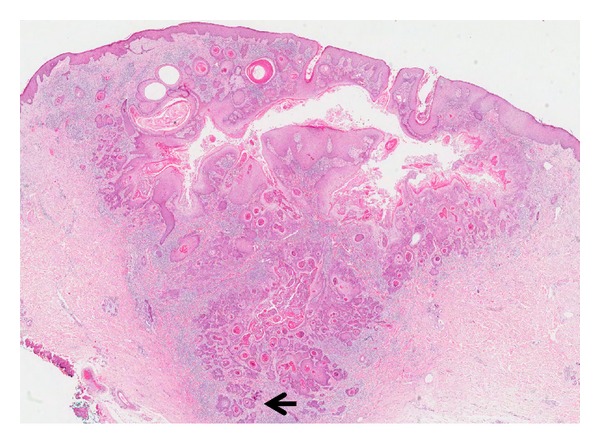
Keratoacanthoma-like SCC: low power magnification showing a crateriform lesion with infiltrative islands and clusters of squamous cells at the base (arrow). (H&E, ×20).

An SCC can sometimes have a crateriform architecture mimicking a KA (KA-like SCC). In this regard, at least part of the tumor should have marked cytologic atypia, frequent or abnormal mitoses, or infiltrative margins (Figures 7 and 16). A shave biopsy that samples only the superficial part of a well-differentiated example of such a lesion could potentially lead to an underdiagnosis of an SCC (Figure 16). Where there is diagnostic doubt about a particular lesion, a complete excision should be recommended. 

### 4.2. Proliferating Tricholemmal Tumor

**Figure 17:**
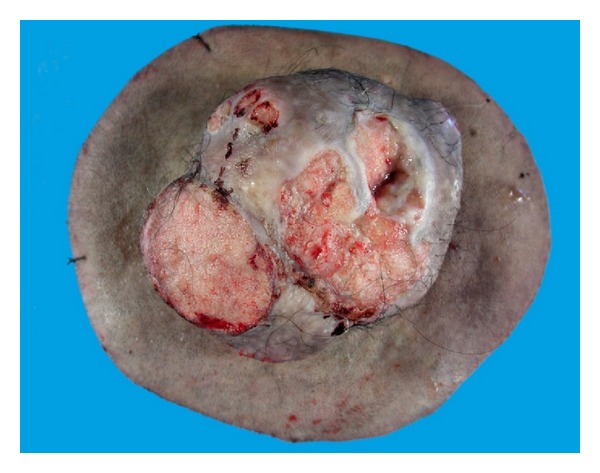
Proliferating tricholemmal tumor: macroscopic photograph showing a circumscribed cutaneous reddish-pink nodular tumor with surface erosions.

**Figure 18:**
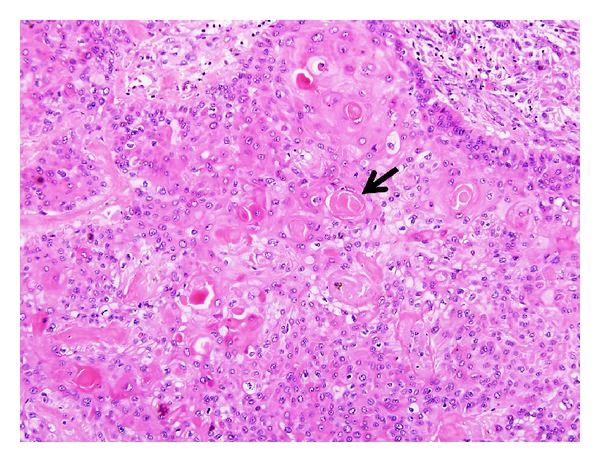
Proliferating tricholemmal tumor: the lesion shows proliferation of squamous cells with clear cytoplasm and abrupt pilar-type keratinization (arrow) (H&E, ×200).

Proliferating tricholemmal tumor (PTT), also known as proliferating pilar tumor or pilar tumor, is a well-circumscribed proliferation of squamous cells [18, 19]. It commonly occurs on the scalp or other parts of the head and neck as a gradually enlarging nodule or tumor (Figure 17). Histopathologically, apart from conventional squamous cells, the tumor shows cellular areas with characteristic clear cytoplasm and abrupt pilar-type keratinization (Figure 18). Calcification and an associated multinucleated cellular reaction are often present. There is a spectrum of appearances which may translate to different biologic behaviors. In a study by Ye et al., tumors that were well differentiated and well circumscribed behaved in a benign fashion [18]. Those with infiltrative borders but no significant atypia, increased mitoses, or necrosis had a local recurrence rate of 15%, while tumors which were infiltrative and which displayed cellular atypia, necrosis, and abnormal mitotic activity had a 50% chance of developing recurrence or metastasis to the regional lymph nodes. The latter category could be termed as a malignant PTT and viewed as equivalent to conventional SCC. Pathological examination of the entire lesion is necessary to assess the borders of the tumor as well as for the possible presence of necrosis and cytologic variation. 

## 5. Bowen Disease (Squamous Cell Carcinoma *In Situ*)

**Figure 19:**
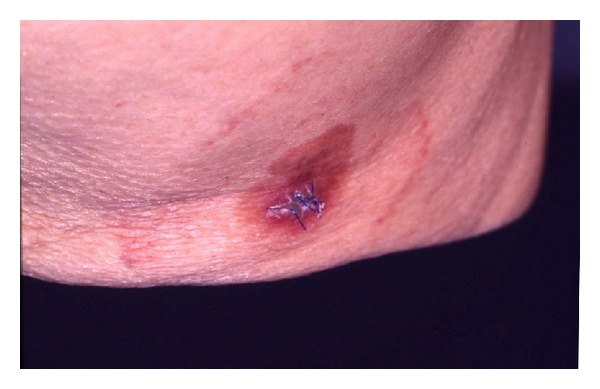
Clinical photograph showing a red, scaly plaque on the skin surface. The patient just had a biopsy performed with sutures still in place.

**Figure 20:**
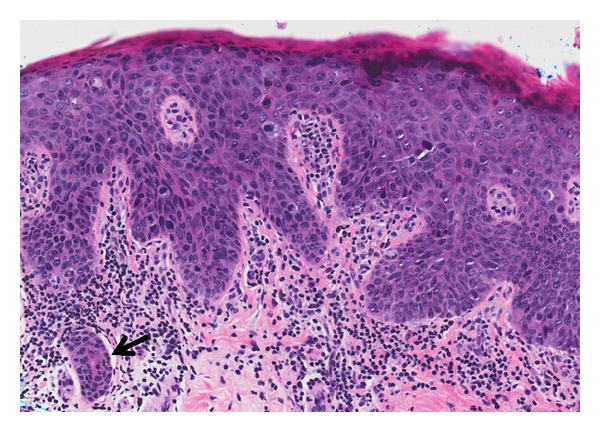
Bowen disease: medium-power magnification showing skin with full thickness epidermal dysplasia which is characterized by loss of the nuclear polarity, nuclear pleomorphism, and mitoses at all levels. The undulating deep epidermal contour and presence of eccrine ducts that have been colonized by the tumor cells (arrow) can potentially mimic invasion. (H&E, ×200).

Bowen disease clinically presents as a plaque in covered as well as uncovered skin areas (Figure 19). Histopathologically, it features full thickness dysplasia, incorporating loss of polarity of the epidermis, nuclear pleomorphism, and mitosis at all levels (Figures 3 and 20). In usual cases, preinvasive malignancy is not in doubt in view of the previously described features. The differential diagnoses classically include extramammary Paget disease and melanoma *in situ* [20]. All three entities display single and groups of atypical cells within the epidermis. The tumor cells of Paget disease generally cluster above an intact basal layer of epidermis and may be demonstrated to contain mucin. Melanoma tumor cells often contain melanin although Paget cells have been known to show this feature as well. Immunohistochemistry helps to distinguish the entities: the tumor cells of Bowen disease are p63 positive, those of Paget's disease react for p-CEA, Ber-EP4, CK7, and CAM5.2, while melanoma cells are positive for S-100 and Melan-A [20]. 

Not uncommonly, Bowen disease shows a markedly undulating basal contour and can be associated with subjacent dermal fibroplasia, raising the possibility of whether early invasion has occurred. The neoplastic epithelium of Bowen disease can also colonize eccrine ducts, potentially exaggerating the incorrect impression of invasion (Figure 20). Examination of deeper levels or more blocks of tissue may reveal foci of convincing invasion, characterized by single or small jagged clusters of infiltrating tumor cells within unequivocally desmoplastic dermal stroma. 

## 6. Other Malignant Tumors That Mimic Primary Cutaneous SCC

### 6.1. Basal Cell Carcinoma

**Figure 21:**
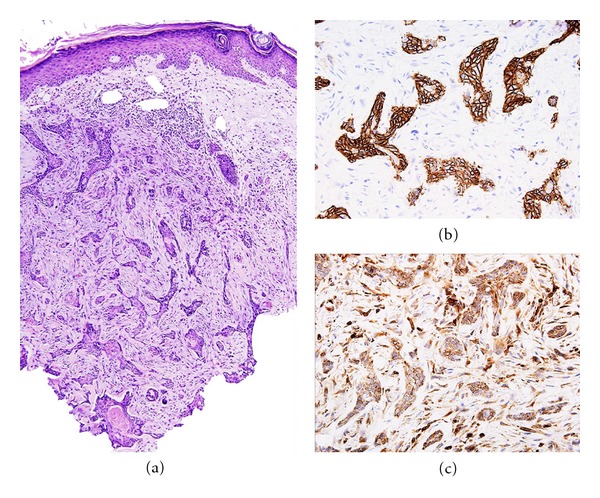
Basal cell carcinoma: (a) low power magnification showing infiltrative irregular islands of basaloid tumor cells which appear to have more ample eosinophilic cytoplasm mimicking the appearance of SCC (H&E, ×100). (b) Tumor cells are diffusely positive for Ber-EP4. (c) Tumor cells also show diffuse positivity for bcl-2.

Basal cell carcinoma (BCC) can sometimes mimic SCC. The typical BCC is characterized by basaloid cellular proliferation with peripheral palisading and tumor-stromal cleft formation. The infiltrating tumor cells may not uncommonly appear squamoid and raise the possibility of SCC. Immunohistochemistry can be helpful as BCC, and not SCC, is positive for Ber-EP4 and bcl-2 (Figure 21) [2]. 

**Figure 22:**
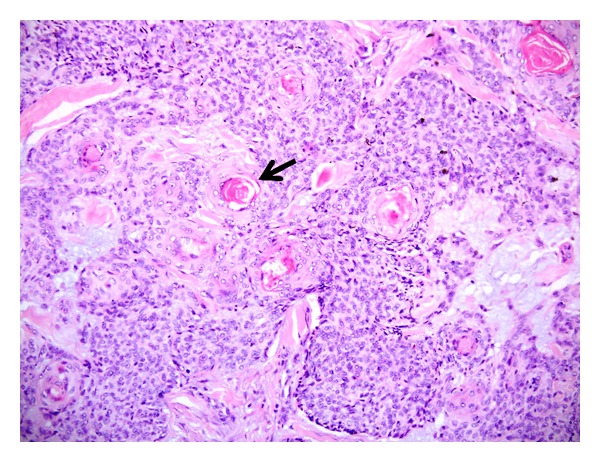
Keratotic BCC: medium-power magnification showing solid growth of tumor cells with foci of squamous differentiation featuring keratin pearls (arrow). Focal tumour-stromal cleft formation with myxoid substance is seen on the right. (H&E, ×200).

The two variants of BCC that have significant squamous components are keratotic BCC (Figure 22) and basosquamous carcinoma (metatypical BCC), the latter having a worse prognosis than BCC in general [21]. A careful assessment of the tumor would usually show areas of more typical BCC.

### 6.2. Sebaceous Carcinoma

The other skin cancer in the head and neck that occasionally mimics a poorly differentiated SCC is sebaceous carcinoma [22]. This tumor is disposed of in sheets and lobules of cells with bubbly cytoplasm that indent an atypical nucleus. Rarely, squamous differentiation may be present [23]. A basic immunochemistry panel can usually enable differentiation between SCC (EMA +ve, Ber-EP4 −ve), sebaceous carcinoma (EMA +ve, Ber-EP4 +ve), and BCC (EMA −ve, Ber-EP4 +ve) [22]. More recently, markers that have been found to be more specific for sebaceous carcinoma include adipophilin and androgen receptor, which in turn are usually negative in BCC and SCC [24].

### 6.3. Melanoma

**Figure 23:**
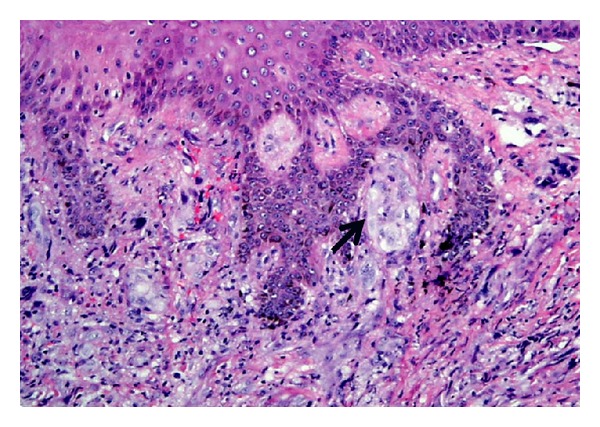
Spindle cell melanoma: medium-power magnification showing dermal pleomorphic spindle cell proliferation and focal junctional melanocytic nest (arrow). (H&E, ×100).

Spindle cell melanoma is one of the classic differential diagnoses for pleomorphic spindle cell tumors of the skin [25]. While cytological features may not enable distinction from a spindle cell SCC and atypical fibroxanthoma (AFX), the presence of a junctional component helps delineate spindle cell melanoma (Figure 23). In contrast, careful examination of a spindle cell SCC may reveal overlying Bowen disease or actinic keratosis. By immunohistochemistry, expression of one or more of the cytokeratins helps distinguish the tumor from spindle cell melanoma which would instead express S-100. AFX is typically S-100 and cytokeratin negative and positive for CD10 and procollagen-1 [26].

### 6.4. Other Carcinomas with Squamous Differentiation

Other skin carcinomas that sometimes feature squamous differentiation include Merkel cell carcinoma (MCC), porocarcinoma, and sebaceous carcinoma [23, 27, 28]. This is likely a manifestation of divergent differentiation of the tumor or varied differentiation of tumor stem cells. Careful examination of MCC would usually show up the more typical malignant small round cells of MCC as well as dot-like expression of CK20 and positivity for the neuroendocrine markers synaptophysin, neurofilament protein, and CD56. As for porocarcinoma, the neoplastic poroid cells should be discernible with duct lumina formation decorated by EMA and p-CEA immunohistochemistry.

### 6.5. Metastatic Squamous Cell Carcinoma to the Skin

**Figure 24:**
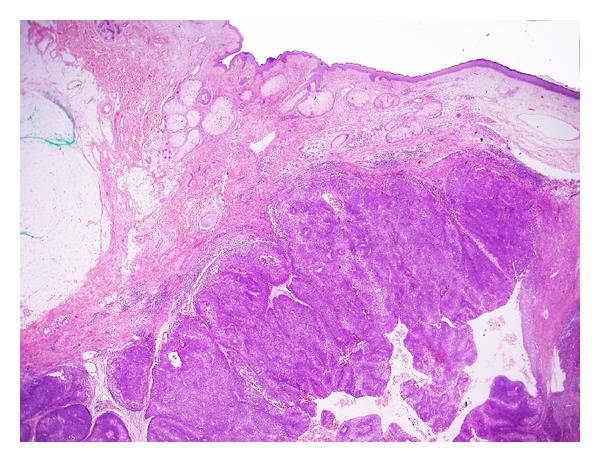
Metastatic SCC: low-power magnification showing solid growth of SCC which is predominantly located in the dermis and subcutaneous tissue. The overlying epidermis appears normal. This should raise suspicion of a metastatic deposit. (H&E, ×40).

An SCC present in the skin may occasionally represent direct invasion from an underlying malignancy or metastatic deposit. In such situations, the importance of clinical history and correlation cannot be overemphasized. The tumor tends to show a centre that is based in the dermis or subcutis (Figure 24). The overlying epidermis tends to show only reactive features or be ulcerated by the growth of the subjacent tumor. 

### 6.6. Lymphoproliferative Disorders

**Figure 25:**
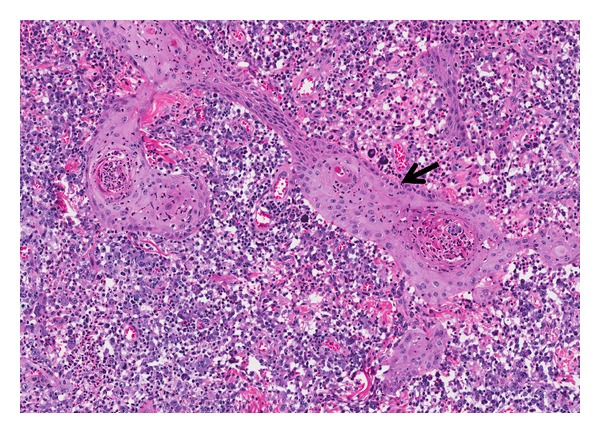
Cutaneous anaplastic large cell lymphoma: low-power magnification showing hyperplastic squamous islands (arrow) associated proliferation of large neoplastic lymphocytes. (H&E, ×100).

Anaplastic large cell lymphoma and lymphomatoid papulosis are CD30-positive lymphoproliferative disorders that have reported cases being associated with epidermal hyperplasia [29]. While the epidermal hyperplasia is often mild in degree, it can be very florid with features of pseudoinfiltration which mimic SCC, raising the possibility of a collision between the carcinoma and lymphoproliferative process (Figure 25). Cellular atypia of the epidermal hyperplasia, while usually absent, has sometimes been reported. Mitoses of the squamous cells, if present, should be seen to be located at the basal layer of the squamous islands and not be abnormal in form. 

## 7. Collision Tumors

SCC can sometimes coexist with other malignant skin tumors within the same tumor mass, the most likely context being collision tumors. Combinations of SCC with melanoma or MCC are most well known [30, 31]. The practical importance is that on finding a prominent SCC component, the coexistence of a potentially more aggressive tumor, or a tumor that may require specific adjuvant therapy, should be sought for. Such a concurrence is not surprising as ultraviolet light exposure is a well known and common risk factor. Another plausible etiologic factor that has emerged more recently is that of human polyomaviruses. While the Merkel cell polyomavirus has been closely linked to MCC, its detection and role in SCC are less consistent and clear [31–33]. Interestingly, the virus has been detected in two cases of combined SCC and MCC [31]. The role of such human polyomaviruses in skin carcinogenesis deserves further investigation. 

## 8. Conclusion

In summary, this review serves to highlight the spectrum of conditions that could mimic SCC: benign, preinvasive, and malignant. Being aware of the pertinent differential diagnoses provides the setting to minimize the pitfalls in the diagnosis of challenging cases which feature squamous proliferation. This, coupled with a systematic assessment for the criteria required for an SCC diagnosis, helps in the rendering of a correct diagnosis. Of the adjunctive studies, immunohistochemistry is the most widely used, helping to indicate the histogenesis of the lesional cells of nonsquamous mimics. In difficult cases, seeking a second opinion provides additional perspectives and insight.

Clinicopathological correlation is of paramount importance in the diagnosis of squamous epithelial lesions. A detailed and relevant history of cancers of the skin and other organs should be sought for. The clinical tempo of the lesion, knowledge of the site, size, and other characteristics are all critical pieces of information to the pathologist. If a lesion is clinically concerning, but the biopsy features are benign or nonspecific, further levels on the block often prove useful. Rarely, a summative assessment of inadequate material or of an atypical lesion that cannot exclude malignancy may have to be given with a recommendation for complete excisional biopsy. As always, this is balanced against the factors of cosmesis and practicability. On the one hand, the final reexcision specimen provides the opportunity for further evaluation of the residual lesion for definite diagnosis. On the other hand, the repeat excision often represents the definite surgical treatment for the lesion if it indeed turns out to be malignant. Such a pragmatic approach offers the rendering of a safe yet clinically useful diagnosis when diagnostically challenging cases are encountered.
